# Neuropathologic Profiles and Associated Cognitive Trajectories in Community-Living Older Adults

**DOI:** 10.1001/jamanetworkopen.2025.54354

**Published:** 2026-01-16

**Authors:** Lei Yu, Tianhao Wang, Lianlian Du, David A. Bennett, Julie A. Schneider, Patricia A. Boyle

**Affiliations:** 1 Rush Alzheimer’s Disease Center, Rush University Medical Center, Chicago, Illinois; 2 Department of Neurological Sciences, Rush University Medical Center, Chicago, Illinois; 3 Department of Pathology, Rush University Medical Center, Chicago, Illinois; 4 Department of Psychiatry and Behavioral Sciences, Rush University Medical Center, Chicago, Illinois

## Abstract

**Importance:**

Mixed neuropathologies are common, and yet the full extent to which these pathologies coexist is not entirely clear.

**Objectives:**

This study aims to identify distinct neuropathologic profiles in community-dwelling older adults and to examine associated cognitive trajectories over time.

**Design, Setting, and Participants:**

This study included participants in 2 community-based cohort studies of aging and dementia. Participants were older adults without known dementia at enrollment who agreed to annual evaluations and brain donation. The Religious Orders Study started in 1994, and the Rush Memory and Aging Project started in 1997. Both studies are ongoing. Data were analyzed in May 2025.

**Exposures:**

Annual uniform detailed evaluations for up to 30 years and brain autopsy and neuropathologic evaluation after death.

**Main Outcomes and Measures:**

Neuropathologic evaluations assessed Alzheimer disease neuropathologic change (ADNC), Lewy bodies, limbic-predominant age-related transactive response DNA-binding protein 43 kDa encephalopathy neuropathologic change (LATE-NC), hippocampal sclerosis, infarcts, cerebral amyloid angiopathy, atherosclerosis, and arteriolosclerosis. Annual cognitive scores were derived from a battery of 19 tests that assessed multiple cognitive domains. Latent neuropathologic profiles were identified using hierarchical clustering, and longitudinal cognitive trajectories associated with each profile were estimated using functional mixed-effects models.

**Results:**

A total of 1633 older adults were included in the study. The mean (SD) age at death was 90.4 (6.4) years, 1156 (70.8%) were female, and the mean (SD) years of education was 16.2 (3.6) years. A total of 46 participants (2.8%) self-reported as Black, 42 (2.6%) as Hispanic, and 1579 (96.6%) as White. More than 80% of individuals had mixed neuropathologies at autopsy, and 280 unique combinations of copathologies were identified. Hierarchical clustering revealed 5 distinct neuropathologic profiles: profile 1 (259 participants [15.9%]) was characterized by a high burden of infarcts and vessel diseases, profile 2 (201 participants [12.3%]) by high LATE-NC and hippocampal sclerosis, profile 3 (355 participants [21.7%]) by high Lewy bodies, profile 4 (159 participants [9.7%]) by high ADNC and amyloid angiopathy, and profile 5 (659 participants [40.4%]) by low pathology overall. Cognitive trajectories differed across profiles in terms of both the rate of decline and the timing of the onset of decline with fastest decline observed for profile 2 and 4.

**Conclusions and Relevance:**

In this cohort study, mixed neuropathologies were extremely common and complex. Compared with vascular conditions, degenerative pathologies clustered into distinct profiles. Profiles characterized by high burdens of ADNC and separately, LATE-NC and hippocampal sclerosis, had the most potent associations with cognitive decline.

## Key Points

**Question:**

What are the latent neuropathologic profiles and associated cognitive trajectories in community-living older adults?

**Findings:**

In this cohort study with 1633 older adults, more than 80% of the individuals had mixed pathologies, with 280 distinct combinations of copathologies. Hierarchical clustering and functional mixed-effects models identified 5 distinct neuropathologic profiles and associated cognitive trajectories.

**Meaning:**

These findings suggest that mixed neuropathologies are complex, some profiles have stronger associations with cognitive aging.

## Teaser

This cohort study describes distinct neuropathologic profiles in the donated brains of older adults and examines associated cognitive trajectories over time..

## Introduction

Compelling evidence from clinicopathologic studies suggest that Alzheimer disease and Alzheimer disease–related dementias (AD/ADRDs) are heterogeneous clinical syndromes attributable to AD and other non-AD neuropathologic conditions.^1,2,3^ Age-related pathologies rarely occur in isolation, and mixed pathologies are commonly observed in aged brains.^4,5,6,7^ The full extent to which these neuropathologies coexist, however, is not entirely clear, as few studies have the relevant data to examine the full complement of age-related neuropathologic indices. We previously reported that more than 200 distinct combinations of copathologies were present in approximately 1000 brains with the 9 most common neuropathologies examined (ie, AD neuropathologic change [ADNC], Lewy bodies, limbic-predominant age-related transactive response DNA-binding protein 43 kDa [TDP-43] encephalopathy neuropathologic change [LATE-NC], hippocampal sclerosis, macroscopic infarcts, microinfarcts, cerebral amyloid angiopathy [CAA], atherosclerosis, and arteriolosclerosis).^8^ In a separate retrospective study of 1600 autopsied individuals, up to 7 pathologies were observed concurrently and resulted in over 160 distinct pathologic combinations.^9^ Furthermore, in a study^10^ of nearly 1500 deceased older adults that examined only cerebrovascular conditions, 80% had cerebrovascular pathologies, and of those, over 60% had vascular copathologies with the most common being vessel diseases mixed with macroscopic infarcts. Of note, prior findings were largely descriptive and specific combinations of copathology observed could have been due to random variations. Advanced statistical techniques facilitate the characterization of distinct profiles of copathologies. A recent study applied item response theory (IRT) to 8 neuropathologic indices including Braak stage, neuritic plaques, Thal stage, Lewy bodies, and TDP-43 in amygdala, limbic and neocortex, as well as hippocampal sclerosis.^11^ Three factors were identified, 1 representing ADNC, 1 representing TDP-43 and hippocampal sclerosis, and 1 representing Lewy bodies. Notably, coexisting cerebrovascular conditions were not considered.

In the current study we analyzed an array of neuropathologic indices designed to capture a wide spectrum of aging-related diseases that affect cognition in more than 1600 community dwelling older adults who were recruited without known dementia, who were followed up annually, and who underwent brain autopsies after death. Using hierarchical clustering, we identified 5 groups of individuals, each representing a unique neuropathologic profile. Furthermore, to investigate the cognitive consequence of these distinct profiles, we examined the corresponding cognitive trajectories using longitudinal data from annual cognitive assessments that spanned up to 30 years before death.

## Methods

### Study Participants

Data came from participants of 1 of the 2 ongoing cohort studies on aging and dementia: the Religious Orders Study (ROS) and the Rush Memory and Aging Project (MAP).^12^ ROS started in 1994 and MAP in 1997, and both studies are ongoing. Participants in ROS are older nuns, priests, and brothers from religious orders throughout the US. Participants in MAP are older adults of more diverse socioeconomic and cultural backgrounds recruited from communities in the Chicago metropolitan area. To facilitate combined analysis, ROS and MAP follow essentially the same study protocol, are conducted by the same investigative team at the Rush Alzheimer’s Disease Center, and share a large common core of testing batteries. All participants enroll without known dementia and are followed up annually until death. Each year, participants undergo a uniform comprehensive evaluation, which includes a medical history interview, detailed cognitive testing, and a neurologic examination (see eMethods in Supplement 1 for details on the sample). The study followed the Strengthening the Reporting of Observational Studies in Epidemiology (STROBE) reporting guideline.

Both studies were approved by the institutional review board of Rush University Medical Center. Before enrollment, each participant signed an informed consent, a separate repository consent to share data and/or biospecimens for research purposes, as well as an Anatomic Gift Act for brain donation.

### Neuropathologic Evaluations

Brain autopsies were performed following a standard procedure, blinded to all clinical data.^13^ The brain was carefully removed, weighed, and bisected. After gross inspection, the hemisphere with more visible pathologies was fixed in 4% paraformaldehyde solution for histological analysis. After fixation for 30 days, the hemisphere was cut into 1-cm coronal slabs and processed for further sectioning and staining. Uniform and systematic neuropathologic evaluations were conducted to assess common neurodegenerative and cerebrovascular conditions in prespecified brain regions.

Pathologic AD diagnosis was determined according to the National Institute on Aging—Alzheimer Association criteria, which requires an intermediate or high AD neuropathologic change.^14^ Separately, continuous measures for β-amyloid and paired helical filament (PHF) tau tangles were obtained using immunohistochemistry (eMethods in Supplement 1).

Lewy bodies were assessed using monoclonal antibodies to phosphorylated α-synuclein (clone LB 509; 1:50; Zymed Labs, Invitrogen Corp; or clone pSyn#64, 1:20 000; Wako Chemicals Inc). Presence of Lewy bodies in 7 brain regions (ie, midfrontal, inferior parietal, middle temporal, cingulate, entorhinal cortices, amygdala, and substantia nigra) was recorded and analyzed as a binary measure.^15^

LATE-NC was assessed using a monoclonal antibody to phosphorylated TDP-43 (pS409/410; 1:100). Semiquantitative measures that capture TDP-43 cytoplasmic inclusions in anterior temporal pole, midfrontal, middle temporal, inferior orbital frontal, entorhinal cortices, hippocampus, and amygdala were summarized into 4 LATE-NC stages, where stage 0 represents no inclusion, stage 1 represents TDP-43 localized to amygdala, stage 2 represents TDP-43 extended to hippocampus or entorhinal cortex, and stage 3 represents extension further to the neocortex.^16^

Hippocampal sclerosis refers to severe neuronal loss and gliosis in CA1 and/or subiculum.^17^ The presence of hippocampal sclerosis was determined using 6-µm hematoxylin and eosin–stained sections from the midhippocampus and analyzed as a binary measure. Multiple cerebrovascular conditions including macroscopic and microinfarcts, CAA, atherosclerosis, and arteriolosclerosis were also assessed (eMethods in Supplement 1).

### Cognitive Assessments

At baseline and annual follow-up evaluations, a uniform comprehensive cognitive testing battery was administered to the study participants. The battery consists of 19 tests that assess multiple relatively distinct cognitive domains. In particular, 7 tests (word list, word list recall, word list recognition, East Boston immediate and delay recall, and logical memory I and II) assess episodic memory, and 4 tests (symbol digits modality, number comparison, Stroop color naming, and word reading) assess perceptual speed. To reduce the ceiling and floor artifacts of individual tests, raw scores for each test were standardized using baseline means and SDs of the entire ROS and MAP cohorts, which were then averaged across all the 19 tests to obtain a global cognitive score.^18^ Higher scores indicate higher cognition. Scores for episodic memory and perceptual speed were computed similarly. After a participant died, a neurologist reviewed all available clinical data and rendered a summary diagnostic opinion of no cognitive impairment, mild cognitive impairment, or dementia.^5^

### Statistical Analysis

Descriptive statistics summarized the characteristics of the study participants. Latent neuropathologic profiles were identified using the hierarchical clustering analysis based on the Ward minimum variance method.^19^ Ten neuropathologic indices (ie, β-amyloid, PHF tau tangles, Lewy bodies, LATE-NC, hippocampal sclerosis, atherosclerosis, arteriolosclerosis, macroscopic infarcts, microinfarcts, and CAA) were used in hierarchical clustering (eFigure 2 in Supplement 1). The optimal number of clusters was determined by following the cluster dendrogram to the point where no meaningful reduction of height could be obtained and verified using Gap statistic (eFigure 3 in Supplement 1).^20^ Model interpretability was also considered. Each resulting cluster represents a group of participants with a distinct neuropathologic profile.

Functional mixed-effects (FME) models were used to estimate and compare the longitudinal cognitive trajectories of each neuropathologic profile. We first examined the trajectories of global cognition, and we also compared the trajectories of episodic memory and perceptual speed. In these models, flexible curves were fit to the annual cognitive data by using B-splines with 5 interior knots equally spaced in quantiles. The knot placement ensures a roughly even distribution of knots relative to the data density and is particularly useful when the data are irregularly observed in a longitudinal study setting.^21^ The number of knots was selected to have 9 cubic B-spline functions, corresponding to a mean (SD) of 9 (5) longitudinal observations per participant. The smoothness penalty terms were estimated using a data-driven restricted maximum likelihood method.^22,23^

All analyses were performed in R version 4.4.2 (R Project for Statistical Computing). The hierarchical clustering analysis was implemented using the R hclust package. The FME models were implemented using the algorithm detailed in our previous publication.^24^ Statistical significance was determined at a nominal level of α = .05. Data were analyzed in May 2025.

## Results

### Characteristics of the Study Participants

Among the 1633 participants included in this study, the mean (SD) age at death was 90.4 (6.4) years. Participants had a mean education of 16.2 (3.6) years, and 1158 (70.8%) were female. A total of 46 participants (2.8%) self-reported as Black, 42 (2.6%) as Hispanic, and 1579 (96.6%) as White. At death, 523 (32.0%) had no cognitive impairment, 380 (23.3%) had mild cognitive impairment, and 729 (44.7%) had dementia.

Brain autopsy was performed a mean (SD) of 9.7 (8.3) hours postmortem. Neuropathologic assessments showed that 1107 participants (67.8%) met the pathologic criteria for AD, 448 (27.4%) had Lewy bodies, 226 (13.8%) had possible or definite hippocampal sclerosis, and about a third (594 participants [36.4%]) had TDP-43 cytoplasmic inclusions extending into hippocampus or entorhinal cortex and beyond. A total of 532 participants (32.5%) had moderate to severe atherosclerosis, and similar percentages (between 32% and 38%) were observed for other vascular conditions (Table 1).

**Table 1.** Characteristics of Study Participants

Characteristic	Participants, No. (%) (N = 1633)
Age at death, mean (SD), y	90.37 (6.41)
Sex	
Female	1156 (70.8)
Male	477 (29.2)
Education, mean (SD), y	16.21 (3.57)
Race and ethnicity^a^	
Black	46 (2.8)
Hispanic	42 (2.6)
White	1577 (96.6)
Other	10 (0.6)
APOE ε4	387 (24.9)
Clinical diagnosis	
NCI	523 (32.0)
MCI	380 (23.3)
Dementia	729 (44.7)
ADNC	1107 (67.8)
β-amyloid load, mean (SD)	1.09 (0.74)
PHF tau tangle density, mean (SD)	1.39 (0.98)
Lewy bodies	448 (27.4)
LATE-NC	
Stage 0	747 (45.7)
Stage 1	292 (17.9)
Stage 2	178 (10.9)
Stage 3	416 (25.5)
Hippocampal sclerosis	226 (13.8)
Atherosclerosis	
None	293 (17.9)
Mild	829 (50.8)
Moderate	415 (25.4)
Severe	96 (5.9)
Arteriolosclerosis	
None	541 (33.1)
Mild	560 (34.3)
Moderate	396 (24.2)
Severe	136 (8.3)
Macroscopic infarcts	
None	1035 (63.4)
1	327 (20.0)
≥2	271 (16.6)
Microinfarcts	
None	1106 (67.7)
1	309 (18.9)
≥2	218 (13.3)
Cerebral amyloid angiopathy	
None	338 (20.7)
Mild	675 (41.3)
Moderate	384 (23.5)
Severe	236 (14.5)

Abbreviations: ADNC, Alzheimer disease neuropathologic change; APOE, apolipoprotein E; LATE-NC, limbic-predominant age-related transactive response DNA-binding protein 43 kDa encephalopathy neuropathologic change; MCI, mild cognitive impairment; NCI, no cognitive impairment; PHF, paired helical filament.

^a^Race was self-reported, and other includes American Indian or Alaska Native, Asian, and Native Hawaiian or other Pacific Islander.

### Latent Neuropathologic Profiles

Notably, a small number of participants had no evidence of neuropathologic conditions at autopsy (84 participants [5.1%]), 115 (7.0%) had only 1 degenerative condition, and 99 (6.1%) had only 1 vascular condition. Consistent with our prior findings,^8^ copathologies were very common. Of the 1335 (81.8%) individuals with mixed pathologies, 124 (9.3%) had mixed vascular pathologies and 104 (7.8%) had mixed degenerative pathologies. The majority of individuals (1107 participants [82.9%]) had both degenerative and vascular pathologies, with 937 (84.6%) having ADNC. In total, 280 distinct copathologies were observed, and the numbers of participants with each distinct combination of pathologies ranged between 1 and 57 (eTable 1 in Supplement 1). With the exception of ADNC with CAA (57 participants), the distinct copathologies were present in less than 3% of deceased participants, reflective of a very complex nature of pathologic findings in aged brains (eFigure 1 in Supplement 1).

**Figure 1.**
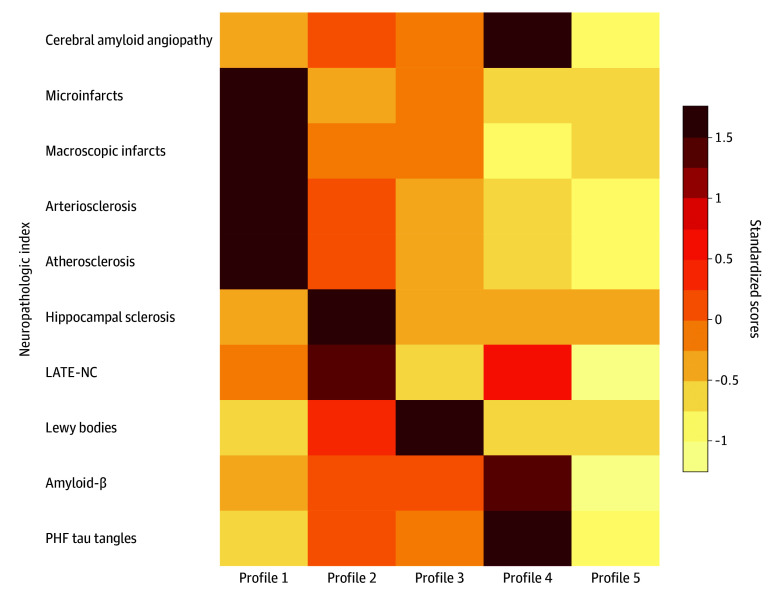
Burdens of Neuropathologies by Profiles The heatmap illustrates the burden (standardized) of the 10 neuropathologic indices across the 5 profiles identified. Darker colors represent higher neuropathologic burdens, and pathologic indices that share similar shades of color tend to cluster together. LATE-NC indicates limbic-predominant age-related transactive response DNA-binding protein 43 kDa encephalopathy neuropathologic change; PHF, paired helical filament.

Results from the hierarchical clustering analysis suggested that a 5-cluster model fit the data best (eResults in Supplement 1). The final clustering results were summarized into 5 latent profiles using standardized scores (Figure 1). Profile 1 (259 participants [15.9%]) consists of individuals with a high burden of infarcts and vessel diseases. Profile 2 (201 participants [12.3%]) consists of individuals with high LATE-NC and hippocampal sclerosis (HS). Profile 3 (355 participants [21.7%]) consists of individuals with high Lewy bodies. Profile 4 (159 participants [9.7%]) consists of individuals with high ADNC and CAA. Profile 5 (659 participants [40.4%]) consists of individuals with a low burden of pathology overall.

Interestingly, despite the difference in latent neuropathologic profiles, demographic characteristics including age, sex, and education were relatively similar across these groups (Table 2). By contrast, we observed a significant difference in clinical diagnosis proximate to death. Profile 5 (those with the relatively low burden of pathology) had the lowest percentage of dementia (270 participants [41.0%]) compared with the other 4 profiles. Profile 5 also had the highest percentage of participants who died with no cognitive impairment (232 participants [35.2%]). Profiles 2 and 4 had the highest proportions of ε4 carriers, while profile 5 had the lowest proportion of ε4 carriers. Results for other common AD/ADRD variants were less clear (eTable 2 in Supplement 1).

**Table 2.** Participants Characteristics by Latent Neuropathologic Profiles

Characteristic	Participants by profile, No. (%)				
	1	2	3	4	5
Age at death, mean (SD), y	89.77 (6.50)	90.38 (6.10)	90.63 (6.22)	90.83 (6.04)	90.36 (6.65)
Sex					
Female	177 (68.3)	146 (72.6)	243 (68.5)	109 (68.6)	481 (73.0)
Male	82 (31.7)	55 (27.4)	112 (31.5)	50 (31.4)	178 (27.0)
Education, mean (SD), y	16.48 (3.65)	15.82 (3.57)	16.11 (3.46)	16.18 (3.77)	16.29 (3.56)
APOE ε4	63 (25.5)	52 (27.2)	89 (26.3)	40 (26.7)	143 (22.8)
Clinical diagnosis					
NCI	85 (32.8)	64 (31.8)	98 (27.6)	44 (27.7)	232 (35.2)
MCI	56 (21.6)	41 (20.4)	88 (24.8)	38 (23.9)	157 (23.8)
Dementia	118 (45.6)	95 (47.3)	169 (47.6)	77 (48.4)	270 (41.0)
β-amyloid load, mean (SD)	1.08 (0.73)	1.27 (0.70)	1.23 (0.76)	1.67 (0.40)	0.82 (0.69)
PHF tau tangle density, mean (SD)	1.20 (0.69)	1.73 (1.08)	1.53 (1.00)	2.79 (1.01)	0.95 (0.60)
Lewy bodies	5 (1.9)	87 (43.3)	352 (99.2)	3 (1.9)	1 (0.2)
LATE-NC					
Stage 0	84 (32.4)	18 (9.0)	170 (47.9)	35 (22.0)	440 (66.8)
Stage 1	48 (18.5)	17 (8.5)	74 (20.8)	23 (14.5)	130 (19.7)
Stage 2	43 (16.6)	20 (10.0)	47 (13.2)	17 (10.7)	51 (7.7)
Stage 3	84 (32.4)	146 (72.6)	64 (18.0)	84 (52.8)	38 (5.8)
Hippocampal sclerosis	7 (2.7)	200 (99.5)	1 (0.3)	16 (10.1)	2 (0.3)
Atherosclerosis					
None	14 (5.4)	24 (11.9)	67 (18.9)	26 (16.4)	162 (24.6)
Mild	93 (35.9)	107 (53.2)	190 (53.5)	91 (57.2)	348 (52.8)
Moderate	117 (45.2)	53 (26.4)	76 (21.4)	40 (25.2)	129 (19.6)
Severe	35 (13.5)	17 (8.5)	22 (6.2)	2 (1.3)	20 (3.0)
Arteriolosclerosis					
None	40 (15.4)	45 (22.4)	122 (34.4)	60 (37.7)	274 (41.6)
Mild	71 (27.4)	82 (40.8)	125 (35.2)	58 (36.5)	224 (34.0)
Moderate	90 (34.7)	61 (30.3)	81 (22.8)	35 (22.0)	129 (19.6)
Severe	58 (22.4)	13 (6.5)	27 (7.6)	6 (3.8)	32 (4.9)
Macroscopic infarcts					
None	37 (14.3)	133 (66.2)	232 (65.4)	133 (83.6)	500 (75.9)
1	94 (36.3)	38 (18.9)	69 (19.4)	15 (9.4)	111 (16.8)
≥2	128 (49.4)	30 (14.9)	54 (15.2)	11 (6.9)	48 (7.3)
Microinfarcts					
None	71 (27.4)	148 (73.6)	235 (66.2)	130 (81.8)	522 (79.2)
1	94 (36.3)	35 (17.4)	67 (18.9)	23 (14.5)	90 (13.7)
≥2	94 (36.3)	18 (9.0)	53 (14.9)	6 (3.8)	47 (7.1)
Cerebral amyloid angiopathy					
None	58 (22.4)	34 (16.9)	66 (18.6)	1 (0.6)	179 (27.2)
Mild	115 (44.4)	74 (36.8)	149 (42.0)	36 (22.6)	301 (45.7)
Moderate	42 (16.2)	59 (29.4)	86 (24.2)	72 (45.3)	125 (19.0)
Severe	44 (17.0)	34 (16.9)	54 (15.2)	50 (31.4)	54 (8.2)

Abbreviations: APOE, apolipoprotein E; LATE-NC, limbic-predominant age-related transactive response DNA-binding protein 43 kDa encephalopathy neuropathologic change; MCI, mild cognitive impairment; NCI, no cognitive impairment; PHF, paired helical filament.

### Cognitive Trajectories by Latent Neuropathologic Profiles

**Figure 2.**
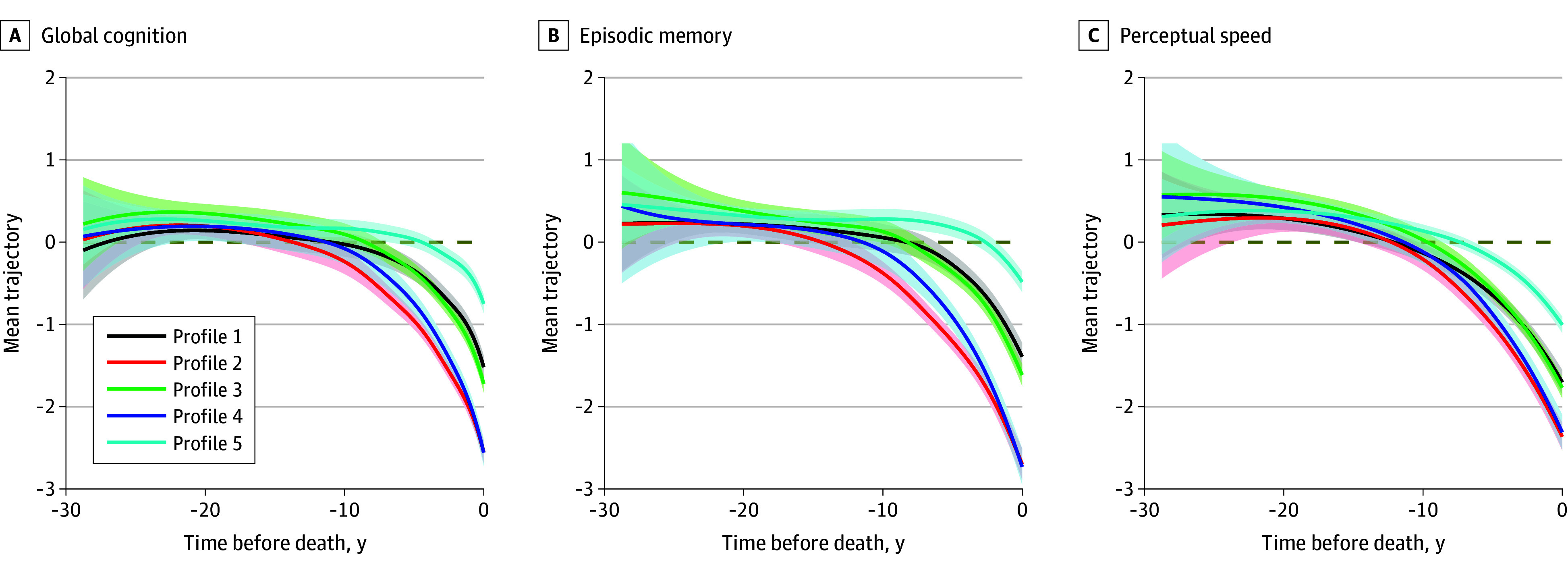
Trajectories of Decline in Global Cognition, Episodic Memory, and Perceptual Speed The figure illustrates the profile-specific trajectories of decline in global cognition, episodic memory, and perceptual speed. The solid lines on each panel are the mean nonlinear trajectories estimated from the functional mixed-effects models, overplotted with the corresponding 95% confidence bands. The dotted horizontal line is the reference line of 0.

To assess the cognitive outcomes of the neuropathologic profiles, we estimated the mean trajectories of longitudinal change in global cognition for each profile (Figure 2). Overall, the cognitive trajectories shared a similar pattern such that decline occurred over a decade before death, and a terminal drop in cognition became evident in the years just before death. The rates of decline, however, varied between the profiles. Profile 5 (low pathologic burden) had the slowest decline, followed by profile 1 (vascular) and profile 3 (Lewy bodies). The fastest decline was observed for profile 2 (LATE-NC and hippocampal sclerosis) and profile 4 (ADNC), with the 2 trajectories tracing one another. The timing of the onset of decline also differed between profiles. Cognitive decline for profile 5 emerged around 10 years before death. In comparison, decline for the other profiles could be observed at least 15 years before death.

Next, considering that trajectories of change in specific cognitive domains could be differentially affected by distinct neuropathologic profiles, we examined the trajectories of episodic memory and separately, perceptual speed (Figure 2). Interestingly, the rates of domain-specific decline by neuropathologic profiles were similar to that of global cognition. Individuals in profile 5 were those with the slowest decline, profiles 2 and 4 had the fastest decline, and profiles 1 and 3 were in between. The distinction between groups was less pronounced for perceptual speed compared with episodic memory. In addition, compared with episodic memory, the decline in perceptual speed occurred much earlier on average.

## Discussion

In this study, we examined common pathologic conditions in autopsied brains from more than 1600 older adults. We observed that over 80% of these individuals had mixed pathologies, with 280 distinct combinations of copathologies. A vast majority of these copathologies involved ADNC, and yet each copathology accounted for only a small proportion of study participants. Hierarchical clustering identified 5 groups of individuals with distinct neuropathologic profiles, and FME models further revealed that cognitive aging trajectories varied between these profiles.

While it has been well acknowledged that mixed pathologies are common in aged brains, the extent to which distinct neuropathologic profiles drive cognitive decline in older adults is not well understood. Prior literature on the neuropathologic correlates of cognitive decline and other functional changes are largely limited to either individual pathologies,^25,26,27^ specific patterns of copathologies (eg, ADNC and LATE-NC),^28,29^ or focusing on older adults with certain neuropathologic conditions (eg, TDP-43 proteinopathy).^30^ To our knowledge, the current study is among the few that have attempted to systematically disentangle complex patterns of copathology and to examine their associations with cognitive trajectories. These findings have several important implications.

First, our data paint a rather complex picture of mixed pathologies in aged brains. Nearly 300 unique combinations of copathologies were observed, and except for ADNC and CAA, each copathology accounted for less than 3% of the overall sample. This vastly heterogenous distribution of copathologies poses a challenge for the ongoing effort to identify potential drug targets or treatment strategies for aging-related cognitive impairment and dementia. Potential interactions of copathologies further complicate the situation. This is exemplified by amyloid-related imaging abnormalities (ARIA) associated with the current antiamyloid treatment.^31^ Complications of ARIA include cerebral microbleeds or intracranial hemorrhage that could be life-threatening. β-Amyloid works as the key driver in the pathogenesis of both AD and CAA, and studies have shown that CAA is a major risk factor for ARIA.^32^ To minimize the ARIA complications, patients with concomitant probable CAA are routinely excluded from antiamyloid immunotherapy for AD. Notably, however, our study suggests that ADNC with CAA is the most common copathology among those observed. This could restrict the applicability of antiamyloid therapy.

Second, the results from our clustering analysis summarized the heterogenous combinations of neuropathologies into 5 profiles. Approximately 40% of individuals had a relatively low neuropathology burden overall, 22% had a high burden of Lewy bodies, 16% had high infarcts and vessel diseases, 12% had high LATE-NC and hippocampal sclerosis, and 10% had high ADNC and CAA. Overall, these classifications are consistent with a prior report based on an IRT application.^11^ We note, however, that not only were the methodologies for identifying these profiles different, but so were the specific neuropathologic indices used for the analysis. Importantly, both studies identified similar groups of neuropathologic profiles that include ADNC, LATE-NC with hippocampal sclerosis, and separately Lewy bodies, lending confidence to the robustness of our findings. In addition, as the current study also included postmortem measures of infarcts and vessel diseases, an additional profile for vascular conditions emerged. In our view, the clusters identified here highlight key neuropathologic contributors for AD/ADRDs. Interestingly, the analysis clustered the major vascular conditions into 1 profile, while each degenerative condition was classified into a separate profile. The grouping of LATE-NC with hippocampal sclerosis was expected, as prior data suggest that there is likely a pathogenetic link between TDP-43 and hippocampal sclerosis.^33,34^ It is worth mentioning that even though individuals from each profile had disproportionately high burdens of specific copathologies (eg, LATE-NC with hippocampal sclerosis for profile 2), they also had fairly substantial burdens of other coexisting pathologies. These results highlight the pervasiveness and extreme complexity of mixed pathologies.

Third, longitudinal cognitive trajectories specific to individual neuropathologic profiles follow a nonlinear pattern and vary both in terms of the rate of decline as well as the timing of the onset of decline. Decline was slowest for the low pathology group (as expected), followed by vascular (profile 1), Lewy bodies (profile 3), and then the LATE-NC and hippocampal sclerosis (profile 2) and ADNC groups (profile 4). These differences suggest that the cognitive consequences are most severe for older adults with ADNC or LATE-NC and hippocampal sclerosis, and less so for Lewy bodies or vascular diseases. This is consistent with several prior findings showing that, relative to vascular conditions, degenerative conditions are the main drivers of cognitive impairment.^3^ In addition, the onset of cognitive decline occurred much later for older adults in the low pathology group compared with those in the high pathology groups. Interestingly, terminal decline occurred on average 2 to 3 years before death across profiles, suggesting that terminal decline is a relatively separate process. Domain-specific findings further suggest that the onset of decline in perceptual speed was consistently earlier than that of memory. This is consistent with our prior reports and the general thinking that decline in perceptual speed occurs relatively early in aging and progresses over time in most older adults.^35^ Interestingly, apart from the rate of decline and timing of the onset, these latent pathology profiles resemble one another in the overall pattern of cognitive decline, and this is even true for select cognitive domains. Future studies are warranted to develop or identify alternative cognitive or other novel noncognitive measures that can qualitatively differentiate older adults with distinct neuropathologic profiles.

Finally, while our data showed that neuropathologies are ubiquitous in the aging brain, approximately a third of deceased participants died with no cognitive impairment. This discordance reinforces the importance of targeting cognitive resilience.^36,37^ A promising aspect of resilience as a therapeutic end point is its potential to offset the insults to cognition from mixed pathologies—a common observation and one that is very difficult to combat. Research focusing on resilience is rapidly expanding, with topics ranging from cortical proteins for neural reserve to modifiable lifestyle and psychosocial factors. Prior evidence suggests that resilience factors are indeed able to prevent cognitive impairment or slow its progression in the presence of any combination of common neuropathologies,^38,39,40,41^ and this remains a fruitful area for further investigation and discovery.

### Strengths and Limitations

This study has many strengths. Participants were community-based, free of known dementia at baseline, and followed annually until death. The design allows for a comprehensive characterization of the natural history of cognitive aging, spanning from normal cognition to mild cognitive impairment and ultimately dementia. Cognitive data were obtained using a comprehensive neuropsychological battery administered at closely spaced intervals, enabling high-fidelity estimation of longitudinal cognitive trajectories. Systematic postmortem evaluations conducted over the past 30 years have generated high-quality and comprehensive data on a wide range of neuropathologic indices associated with AD/ADRDs in a large sample. These data enable a robust, systematic analysis of pathology profiles with high confidence. Importantly, clinical evaluations are administered during annual home visits, which reduces the burden for study participation. As a result, both ROS and MAP have over 95% follow-up rates among the survivors. In addition, brain donation is required for enrollment, which further reduces the dropout due to autopsy consent.

Limitations include a focus on neuropathologic indices most commonly associated with cognitive impairment and dementia. Data collection for less common indices is ongoing. We also acknowledge that not all the pathologies are quantified with the same fidelity. Certain pathologic conditions are rated as present vs absent, which fails to account for the pathologic burden. Separately, participation in ROS and MAP requires brain donation, and participants are predominantly non-Hispanic White individuals with high levels of education who died at very old age. Our findings need to be interpreted in the context of the selective nature of research participants and may not be generalizable to more diverse or the general aging population.

A recent study that combined data from 13 community- and population-based cohorts shows that the frequency of LATE-NC ranges between 11% and 63%. In the current study, the frequency of LATE-NC is toward the upper end. Relative to the studies in the prior report, participants in ROS and MAP were older with a mean age of death over 90. It was previously reported that LATE-NC tends to have higher prevalence among those with more advanced age,^42^ and hence the older age of participants as well as the difference in TDP-43 antibodies might contribute to a higher frequency of LATE-NC reported in this study.

## Conclusions

In this cohort study, we observed that mixed neuropathologies were common and complex such that over 80% of the individuals had mixed pathologies, with 280 distinct combinations of copathologies. Hierarchical clustering and functional mixed-effects models identified 5 distinct neuropathologic profiles, and the profiles characterized by high burdens of ADNC and separately LATE-NC and hippocampal sclerosis had the most potent association with cognitive decline.

## Notes

Supplement 1. eMethods. eReferences. eResults. eTable 1. Frequency of Copathologies of the Study Participants eTable 2. Common AD/ADRD Genetic Variants (Dosage) by Latent Neuropathologic Profiles eFigure 1. Burden of Copathologies of the Study Participants eFigure 2. Hierarchical Cluster Dendrogram eFigure 3. Gap Statistic for Optimal Number of Clusters

Supplement 2. Data Sharing Statement

## References

[1] Power MC, Mormino E, Soldan A, . Combined neuropathological pathways account for age-related risk of dementia. Ann Neurol. 2018;84(1):10-22. doi:10.1002/ana.25246 29944741 PMC6119518

[2] Brookmeyer R, Kawas CH, Abdallah N, Paganini-Hill A, Kim RC, Corrada MM. Impact of interventions to reduce Alzheimer’s disease pathology on the prevalence of dementia in the oldest-old. Alzheimers Dement. 2016;12(3):225-232. doi:10.1016/j.jalz.2016.01.004 26900132 PMC4808364

[3] Boyle PA, Yu L, Leurgans SE, . Attributable risk of Alzheimer’s dementia attributed to age-related neuropathologies. Ann Neurol. 2019;85(1):114-124. doi:10.1002/ana.25380 30421454 PMC10128614

[4] Coulthard EJ, Love S. A broader view of dementia: multiple co-pathologies are the norm. Brain. 2018;141(7):1894-1897. doi:10.1093/brain/awy153 30053176

[5] Schneider JA, Arvanitakis Z, Bang W, Bennett DA. Mixed brain pathologies account for most dementia cases in community-dwelling older persons. Neurology. 2007;69(24):2197-2204. doi:10.1212/01.wnl.0000271090.28148.24 17568013

[6] Schneider JA. Neuropathology of dementia disorders. Continuum (Minneap Minn). 2022;28(3):834-851. doi:10.1212/CON.0000000000001137 35678405 PMC10278955

[7] Kovacs GG, Milenkovic I, Wöhrer A, . Non-Alzheimer neurodegenerative pathologies and their combinations are more frequent than commonly believed in the elderly brain: a community-based autopsy series. Acta Neuropathol. 2013;126(3):365-384. doi:10.1007/s00401-013-1157-y 23900711

[8] Boyle PA, Yu L, Wilson RS, Leurgans SE, Schneider JA, Bennett DA. Person-specific contribution of neuropathologies to cognitive loss in old age. Ann Neurol. 2018;83(1):74-83. doi:10.1002/ana.25123 29244218 PMC5876116

[9] Robinson JL, Xie SX, Baer DR, . Pathological combinations in neurodegenerative disease are heterogeneous and disease-associated. Brain. 2023;146(6):2557-2569. doi:10.1093/brain/awad059 36864661 PMC10232273

[10] Lamar M, Leurgans S, Kapasi A, . Complex profiles of cerebrovascular disease pathologies in the aging brain and their relationship with cognitive decline. Stroke. 2022;53(1):218-227. doi:10.1161/STROKEAHA.121.034814 34601898 PMC8712368

[11] Katsumata Y, Fardo DW, Shade LMP, ; Alzheimer’s Disease Neuroimaging Initiative; National Alzheimer’s Coordinating Center. Genetic associations with dementia-related proteinopathy: application of item response theory. Alzheimers Dement. 2024;20(4):2906-2921. doi:10.1002/alz.13741 38460116 PMC11032554

[12] Bennett DA, Buchman AS, Boyle PA, Barnes LL, Wilson RS, Schneider JA. Religious orders study and rush memory and aging project. J Alzheimers Dis. 2018;64(s1):S161-S189. doi:10.3233/JAD-179939 29865057 PMC6380522

[13] Schneider JA, Wilson RS, Cochran EJ, . Relation of cerebral infarctions to dementia and cognitive function in older persons. Neurology. 2003;60(7):1082-1088. doi:10.1212/01.WNL.0000055863.87435.B2 12682310

[14] Hyman BT, Phelps CH, Beach TG, . National Institute on Aging-Alzheimer’s Association guidelines for the neuropathologic assessment of Alzheimer’s disease. Alzheimers Dement. 2012;8(1):1-13. doi:10.1016/j.jalz.2011.10.007 22265587 PMC3266529

[15] Schneider JA, Arvanitakis Z, Yu L, Boyle PA, Leurgans SE, Bennett DA. Cognitive impairment, decline and fluctuations in older community-dwelling subjects with Lewy bodies. Brain. 2012;135(Pt 10):3005-3014. doi:10.1093/brain/aws234 23065790 PMC3470712

[16] Nag S, Barnes LL, Yu L, Wilson RS, Bennett DA, Schneider JA. Limbic-predominant age-related TDP-43 encephalopathy in Black and White decedents. Neurology. 2020;95(15):e2056-e2064. doi:10.1212/WNL.0000000000010602 32759188 PMC7713750

[17] Nag S, Yu L, Capuano AW, . Hippocampal sclerosis and TDP-43 pathology in aging and Alzheimer disease. Ann Neurol. 2015;77(6):942-952. doi:10.1002/ana.24388 25707479 PMC4447563

[18] Wilson RS, Boyle PA, Yu L, . Temporal course and pathologic basis of unawareness of memory loss in dementia. Neurology. 2015;85(11):984-991. doi:10.1212/WNL.0000000000001935 26311746 PMC4567465

[19] Murtagh F, Legendre P. Ward’s hierarchical agglomerative clustering method: which algorithms implement Ward’s criterion? J Classif. 2014;31:274-295. doi:10.1007/s00357-014-9161-z

[20] Tibshirani R, Walther G, Hastie T. Estimating the number of clusters in a data set via the gap statistic. J R Stat Soc B. 2001;63(2):411-423. doi:10.1111/1467-9868.00293

[21] Ruppert D. Selecting the number of knots for penalized splines. J Comput Graph Stat. 2002;11(4):735-757. doi:10.1198/106186002853

[22] Wahba G. A comparison of GCV and GML for choosing the smoothing parameter in the generalized spline smoothing problem. Ann Stat. 1985;13(4):1378-1402. doi:10.1214/aos/1176349743

[23] Guo W. Functional mixed effects models. Biometrics. 2002;58(1):121-128. doi:10.1111/j.0006-341X.2002.00121.x 11890306

[24] Wang T, Yu L, Leurgans SE, Wilson RS, Bennett DA, Boyle PA. Conditional functional clustering for longitudinal data with heterogeneous nonlinear patterns. Ann Appl Stat. 2022;16(2):1191-1214. doi:10.1214/21-AOAS1542

[25] Karanth SD, Schmitt FA, Nelson PT, . Four common late-life cognitive trajectories patterns associate with replicable underlying neuropathologies. J Alzheimers Dis. 2021;82(2):647-659. doi:10.3233/JAD-210293 34057090 PMC8316292

[26] Liampas I, Dimitriou N, Siokas V, Messinis L, Nasios G, Dardiotis E. Cognitive trajectories preluding the onset of different dementia entities: a descriptive longitudinal study using the NACC database. Aging Clin Exp Res. 2024;36(1):119. doi:10.1007/s40520-024-02769-9 38780681 PMC11116253

[27] LaCroix AZ, Hubbard RA, Gray SL, . Trajectories of physical function prior to death and brain neuropathology in a community-based cohort: the act study. BMC Geriatr. 2017;17(1):258. doi:10.1186/s12877-017-0637-7 29096630 PMC5667523

[28] Thomas DX, Bajaj S, McRae-McKee K, Hadjichrysanthou C, Anderson RM, Collinge J. Association of TDP-43 proteinopathy, cerebral amyloid angiopathy, and Lewy bodies with cognitive impairment in individuals with or without Alzheimer’s disease neuropathology. Sci Rep. 2020;10(1):14579. doi:10.1038/s41598-020-71305-2 32883971 PMC7471113

[29] Kapasi A, Yu L, Boyle PA, Barnes LL, Bennett DA, Schneider JA. Limbic-predominant age-related TDP-43 encephalopathy, ADNC pathology, and cognitive decline in aging. Neurology. 2020;95(14):e1951-e1962. doi:10.1212/WNL.0000000000010454 32753441 PMC7682843

[30] Katsumata Y, Abner EL, Karanth S, . Distinct clinicopathologic clusters of persons with TDP-43 proteinopathy. Acta Neuropathol. 2020;140(5):659-674. doi:10.1007/s00401-020-02211-0 32797255 PMC7572241

[31] Greenberg SM, Bax F, van Veluw SJ. Amyloid-related imaging abnormalities: manifestations, metrics and mechanisms. Nat Rev Neurol. 2025;21(4):193-203. doi:10.1038/s41582-024-01053-8 39794509

[32] Sveikata L, Charidimou A, Viswanathan A. Vessels sing their ARIAs: the role of vascular amyloid in the age of aducanumab. Stroke. 2022;53(1):298-302. doi:10.1161/STROKEAHA.121.036873 34905943

[33] Dickson DW, Baker M, Rademakers R. Common variant in GRN is a genetic risk factor for hippocampal sclerosis in the elderly. Neurodegener Dis. 2010;7(1-3):170-174. doi:10.1159/000289231 20197700 PMC2859236

[34] Yang HS, Yu L, White CC, . Evaluation of TDP-43 proteinopathy and hippocampal sclerosis in relation to APOE ε4 haplotype status: a community-based cohort study. Lancet Neurol. 2018;17(9):773-781. doi:10.1016/S1474-4422(18)30251-5 30093249 PMC6154505

[35] Wilson RS, Segawa E, Hizel LP, Boyle PA, Bennett DA. Terminal dedifferentiation of cognitive abilities. Neurology. 2012;78(15):1116-1122. doi:10.1212/WNL.0b013e31824f7ff2 22491858 PMC3320052

[36] Bennett DA. Mixed pathologies and neural reserve: Implications of complexity for Alzheimer disease drug discovery. PLoS Med. 2017;14(3):e1002256. doi:10.1371/journal.pmed.1002256 28291788 PMC5349649

[37] Negash S, Wilson RS, Leurgans SE, . Resilient brain aging: characterization of discordance between Alzheimer’s disease pathology and cognition. Curr Alzheimer Res. 2013;10(8):844-851. doi:10.2174/15672050113109990157 23919768 PMC4060425

[38] Buchman AS, Yu L, Klein HU, . Glycoproteome-wide discovery of cortical glycoproteins that may provide cognitive resilience in older adults. Neurology. 2024;102(7):e209223. doi:10.1212/WNL.0000000000209223 38502899 PMC11770689

[39] Yu L, Hsieh YC, Pearse RV, . Association of AK4 protein from stem cell-derived neurons with cognitive reserve: an autopsy study. Neurology. 2022;99(20):e2264-e2274. doi:10.1212/WNL.0000000000201120 35948448 PMC9694839

[40] Gravitz L. Drawing on the brain’s resilience to fight Alzheimer’s disease. Nature. 2018;559(7715):S8-S9. doi:10.1038/d41586-018-05720-x 30046079

[41] Yu L, Tasaki S, Schneider JA, . Cortical proteins associated with cognitive resilience in community-dwelling older persons. JAMA Psychiatry. 2020;77(11):1172-1180. doi:10.1001/jamapsychiatry.2020.1807 32609320 PMC7330835

[42] Nelson PT, Dickson DW, Trojanowski JQ, . Limbic-predominant age-related TDP-43 encephalopathy (LATE): consensus working group report. Brain. 2019;142(6):1503-1527. doi:10.1093/brain/awz099 31039256 PMC6536849

