# Supplementary material for: Neuropathologic Profiles and Associated Cognitive Trajectories in Community-Living Older Adults
Source: JAMA Netw Open. 2026 Jan 16;9(1):e2554354. doi: 10.1001/jamanetworkopen.2025.54354 (PMC12811812; doi:10.1001/jamanetworkopen.2025.54354)
Supplement: Supplement 1. — eMethods. eReferences. eResults. eTable 1. Frequency of Copathologies of the Study Participants eTable 2. Common AD/ADRD Genetic Variants (Dosage) by Latent Neuropathologic Profiles eFigure 1. Burden of Copathologies of the Study Participants eFigure 2. Hierarchical Cluster Dendrogram eFigure 3. Gap Statistic for Optimal Number of Clusters [file jamanetwopen-e2554354-s001.pdf]

Supplemental Online Content

Yu L, Wang T, Du L, Bennett DA, Schneider JA, Boyle PA. Neuropathologic profiles and associated cognitive trajectories in community-living older adults. *JAMA Netw Open*. 2026;9(1):e2554354. doi:10.1001/jamanetworkopen.2025.54354

eMethods.

eResults.

eReferences.

eTable 1. Frequency of Copathologies of the Study Participants

eTable 2. Common AD/ADRD Genetic Variants (Dosage) by Latent Neuropathologic Profiles

eFigure 1. Burden of Copathologies of the Study Participants

eFigure 2. Hierarchical Cluster Dendrogram

eFigure 3. Gap Statistic for Optimal Number of Clusters

This supplemental material has been provided by the authors to give readers additional information about their work.

© 2026 Yu L et al. *JAMA Network Open*.

eMethods.

Sample of study participants

The data freeze for these analyses occurred in January 2025; at that time, there were 4,030 older adults enrolled in ROS and MAP. Of the 3,881 participants who completed the baseline evaluation, 2,445 died. Of the deceased, 2,098 had undergone brain autopsies (autopsy rate of 86%), and 2,058 had neuropathologic evaluations approved by a board certified neuropathologist. For the current study, ten postmortem neuropathologic indices were examined, namely β-amyloid, paired helical filaments (PHF) tau tangles, Lewy bodies, LATE-NC, hippocampal sclerosis, macroscopic infarcts, microinfarcts, CAA, atherosclerosis, and arteriolosclerosis. We excluded 287 participants with other major neuropathologic findings (e.g., brain tumor or traumatic brain injury). Of the remaining 1,771, 138 (7.8%) participants without complete neuropathology data were not included in the analysis, leaving a final analytic sample of N=1,633.

Quantification of β-amyloid load and PHF tau tangle density

For β-amyloid, 6μm sections from midfrontal, middle temporal, and inferior parietal cortices, hippocampus, basal ganglia, substantia nigra, and cerebellum were stained using one of the three monoclonal antibodies, 4G8 (1:9000; Covance Labs, Madison, WI), 6F/3D (1:50; Dako North America Inc., Carpinteria, CA), or 10D5 (1:600; Elan Pharmaceuticals, San Francisco, CA). Prior to 2019, β-amyloid was quantified manually using systematic random sampling and image analysis[1]. Beginning in 2019, stained slides were digitally scanned using the Aperio AT2 whole slide brightfield scanning system and analyzed using the Aperio Image Analysis Toolbox Positive Pixel Count algorithm[2]. For each of the above-mentioned 8 regions, the percent area occupied by β-amyloid was scored. After square root transformation to correct for skewness, region-specific scores were averaged to obtain a composite measure for β-amyloid load. Quantification of PHF tau tangles follows a similar protocol. Region-specific tangle density (per mm^2) was obtained using images of AT8 (1:2000, Thermoscientific) stained sections, square root transformed and then averaged across the 8 regions to compute a composite tangle measure.

For both β-amyloid and tangles, the historical data and new digital data were harmonized using a statistical approach, as previously described[2]. Briefly, harmonization was conducted using 413 whole-slide images (WSI) for β-amyloid and 639 WSI for tau tangles. Since the same approach was used for β-amyloid and separately tau tangles, we describe the harmonization for β-amyloid. For each WSI, β-amyloid load was scored using both historical and digital quantification algorithms. After checking for skewness and outliers, historical and digital β-amyloid scores were compared using scatterplots and Pearson correlations. Upon confirming a strong Pearson correlation between the two methods, we fitted linear regression model using the historical amyloid score as the predictor and the digital score as the dependent variable. Harmonized β-amyloid scores were constructed as follows: if a digital score was available, that

© 2026 Yu L et al. *JAMA Network Open*.

value was used directly; otherwise, for the cases with only historical pathology data, regression coefficients from the abovementioned regression model were used to impute the harmonized scores. We applied the same approach to the tau-tangle pathology.

Assessment of cerebrovascular conditions

Multiple cerebrovascular conditions were assessed. Cerebral arteries and their proximal branches at the Circle of Willis were visually inspected for atherosclerosis[3]. Vessels of the anterior basal ganglia were examined for arteriolosclerosis[4]. The β-amyloid depositions in meningeal and parenchymal vessels of midfrontal, middle temporal, inferior parietal, and calcarine were assessed for CAA[5]. Atherosclerosis, arteriolosclerosis, and CAA were each rated using a semi-quantitative scale of none, mild, moderate, and severe. Chronic macroscopic infarcts were recorded during the gross examination and confirmed histologically[6]. Chronic microinfarcts were identified under microscope for a minimum of 9 regions[3]. In the current analyses, a 3-level semi-quantitative measure (no infarct, 1 infarct, and multiple infarcts) was used for scoring macroscopic infarcts and microinfarcts separately.

eResults

We describe the pattern of clustering by branching the analytic sample four times (eFigure 2). The first branching separates participants into two groups by Lewy bodies, LATE-NC, and hippocampal sclerosis. The second branching separates the group with high Lewy bodies, LATE-NC, and hippocampal sclerosis into a subgroup with high Lewy bodies and a subgroup with high LATE-NC and HS. The third branching separates the group of low Lewy bodies, LATE-NC, and hippocampal sclerosis into a subgroup with high ADNC and vascular pathologies and a subgroup with low pathologies across all 10 indices. The fourth branching further separates the group of high ADNC and vascular pathologies into a subgroup of high ADNC and a subgroup of high vascular pathologies. The choice of 5 clusters was supported by Gap statistic (eFigure 3).

© 2026 Yu L et al. *JAMA Network Open*.

eReferences.

1. Bennett DA, Schneider JA, Wilson RS, Bienias JL, Arnold SE. Neurofibrillary tangles mediate the association of amyloid load with clinical Alzheimer disease and level of cognitive function. *Arch Neurol.* 2004;61(3):378-384.
2. Kapasi A, Poirier J, Hedayat A, et al. High-throughput digital quantification of Alzheimer disease pathology and associated infrastructure in large autopsy studies. *Journal of neuropathology and experimental neurology.* 2023;82(12):976-986.
3. Arvanitakis Z, Capuano AW, Leurgans SE, Buchman AS, Bennett DA, Schneider JA. The Relationship of Cerebral Vessel Pathology to Brain Microinfarcts. *Brain pathology (Zurich, Switzerland).* 2017;27(1):77-85.
4. Buchman AS, Leurgans SE, Nag S, Bennett DA, Schneider JA. Cerebrovascular disease pathology and parkinsonian signs in old age. *Stroke.* 2011;42(11):3183-3189.
5. Yu L, Boyle PA, Nag S, et al. APOE and cerebral amyloid angiopathy in community-dwelling older persons. *Neurobiology of aging.* 2015;36(11):2946-2953.
6. Schneider JA, Wilson RS, Bienias JL, Evans DA, Bennett DA. Cerebral infarctions and the likelihood of dementia from Alzheimer disease pathology. *Neurology.* 2004;62(7):1148-1155.

© 2026 Yu L et al. *JAMA Network Open*.

eTable 1. Frequency of Copathologies of the Study Participants

Obs	ADNC	LB	LATE	HS	Macroscopic infarcts	Microinfarcts	CAA	Atherosclerosis	Arteriolosclerosis	N	%
1	0	0	0	0	0	0	1	1	1	1	0.07491
2	0	0	0	1	0	1	1	0	0	1	0.07491
3	0	0	0	1	1	1	1	0	1	1	0.07491
4	0	0	1	0	0	1	1	0	0	1	0.07491
5	0	0	1	0	1	0	1	0	0	1	0.07491
6	0	0	1	0	1	0	1	0	1	1	0.07491
7	0	0	1	0	1	0	1	1	1	1	0.07491
8	0	0	1	0	1	1	1	0	1	1	0.07491
9	0	0	1	0	1	1	1	1	1	1	0.07491
10	0	0	1	1	0	0	1	1	0	1	0.07491
11	0	0	1	1	0	1	1	0	0	1	0.07491
12	0	0	1	1	0	1	1	1	0	1	0.07491
13	0	0	1	1	1	0	0	0	0	1	0.07491
14	0	0	1	1	1	0	1	0	0	1	0.07491
15	0	0	1	1	1	1	0	1	1	1	0.07491
16	0	1	0	0	0	0	1	1	0	1	0.07491
17	0	1	0	0	0	1	0	0	1	1	0.07491
18	0	1	0	0	0	1	1	1	0	1	0.07491
19	0	1	0	0	0	1	1	1	1	1	0.07491
20	0	1	0	0	1	0	0	0	1	1	0.07491
21	0	1	0	0	1	0	1	0	0	1	0.07491
22	0	1	0	0	1	0	1	1	1	1	0.07491
23	0	1	0	1	1	1	0	1	0	1	0.07491
24	0	1	1	0	0	0	0	0	1	1	0.07491
25	0	1	1	0	0	0	1	0	1	1	0.07491
26	0	1	1	0	0	1	0	0	0	1	0.07491
27	0	1	1	0	0	1	0	0	1	1	0.07491
28	0	1	1	0	0	1	1	1	0	1	0.07491
29	0	1	1	0	1	0	0	1	0	1	0.07491
30	0	1	1	0	1	0	1	0	0	1	0.07491
31	0	1	1	0	1	0	1	1	0	1	0.07491

© 2026 Yu L et al. *JAMA Network Open*.

Obs	ADNC	LB	LATE	HS	Macroscopic infarcts	Microinfarcts	CAA	Atherosclerosis	Arteriolosclerosis	N	%
32	0	1	1	0	1	0	1	1	1	1	0.07491
33	0	1	1	0	1	1	0	0	0	1	0.07491
34	0	1	1	1	0	0	0	1	0	1	0.07491
35	0	1	1	1	0	0	1	0	0	1	0.07491
36	0	1	1	1	0	1	0	0	1	1	0.07491
37	0	1	1	1	0	1	0	1	0	1	0.07491
38	0	1	1	1	1	1	0	1	0	1	0.07491
39	1	0	0	1	0	0	0	0	0	1	0.07491
40	1	0	0	1	0	0	1	0	0	1	0.07491
41	1	0	0	1	0	1	1	1	0	1	0.07491
42	1	0	0	1	1	0	0	0	1	1	0.07491
43	1	0	0	1	1	0	0	1	1	1	0.07491
44	1	0	0	1	1	0	1	1	0	1	0.07491
45	1	0	0	1	1	1	1	0	0	1	0.07491
46	1	0	1	0	1	0	1	0	1	1	0.07491
47	1	0	1	1	0	0	0	0	1	1	0.07491
48	1	0	1	1	0	0	0	1	1	1	0.07491
49	1	0	1	1	0	1	0	0	0	1	0.07491
50	1	0	1	1	1	0	0	1	1	1	0.07491
51	1	0	1	1	1	0	1	1	0	1	0.07491
52	1	0	1	1	1	1	0	0	0	1	0.07491
53	1	0	1	1	1	1	0	1	0	1	0.07491
54	1	0	1	1	1	1	1	0	0	1	0.07491
55	1	0	1	1	1	1	1	1	1	1	0.07491
56	1	1	0	0	0	1	1	1	0	1	0.07491
57	1	1	0	1	0	0	1	1	0	1	0.07491
58	1	1	0	1	0	0	1	1	1	1	0.07491
59	1	1	0	1	1	1	1	0	0	1	0.07491
60	1	1	0	1	1	1	1	0	1	1	0.07491
61	1	1	1	0	0	0	1	1	0	1	0.07491
62	1	1	1	0	0	1	0	0	1	1	0.07491
63	1	1	1	0	0	1	1	0	1	1	0.07491
64	1	1	1	0	1	0	0	0	1	1	0.07491

© 2026 Yu L et al. *JAMA Network Open*.

Obs	ADNC	LB	LATE	HS	Macroscopic infarcts	Microinfarcts	CAA	Atherosclerosis	Arteriolosclerosis	N	%
65	1	1	1	0	1	0	1	0	0	1	0.07491
66	1	1	1	0	1	0	1	0	1	1	0.07491
67	1	1	1	0	1	0	1	1	1	1	0.07491
68	1	1	1	0	1	1	0	0	0	1	0.07491
69	1	1	1	1	0	0	1	0	1	1	0.07491
70	1	1	1	1	0	1	0	0	0	1	0.07491
71	1	1	1	1	0	1	0	1	0	1	0.07491
72	1	1	1	1	0	1	1	0	0	1	0.07491
73	1	1	1	1	0	1	1	1	0	1	0.07491
74	1	1	1	1	0	1	1	1	1	1	0.07491
75	1	1	1	1	1	0	0	1	1	1	0.07491
76	1	1	1	1	1	0	1	1	1	1	0.07491
77	1	1	1	1	1	1	1	0	1	1	0.07491
78	1	1	1	1	1	1	1	1	0	1	0.07491
79	1	1	1	1	1	1	1	1	1	1	0.07491
80	0	0	0	0	1	0	1	1	0	2	0.14981
81	0	0	0	1	0	1	0	0	0	2	0.14981
82	0	0	1	0	0	0	1	0	1	2	0.14981
83	0	0	1	0	0	1	0	1	0	2	0.14981
84	0	0	1	0	1	0	0	0	1	2	0.14981
85	0	0	1	1	0	0	0	1	0	2	0.14981
86	0	0	1	1	0	0	1	0	0	2	0.14981
87	0	0	1	1	0	1	0	0	0	2	0.14981
88	0	0	1	1	1	0	0	1	1	2	0.14981
89	0	0	1	1	1	1	0	1	0	2	0.14981
90	0	1	0	0	0	0	1	0	0	2	0.14981
91	0	1	0	0	0	1	0	1	1	2	0.14981
92	0	1	0	0	0	1	1	0	0	2	0.14981
93	0	1	0	0	1	0	0	1	0	2	0.14981
94	0	1	0	0	1	1	0	1	0	2	0.14981
95	0	1	0	0	1	1	1	0	0	2	0.14981
96	0	1	1	0	1	0	0	0	0	2	0.14981
97	0	1	1	1	1	0	0	0	0	2	0.14981

© 2026 Yu L et al. *JAMA Network Open*.

Obs	ADNC	LB	LATE	HS	Macroscopic infarcts	Microinfarcts	CAA	Atherosclerosis	Arteriolosclerosis	N	%
**98**	0	1	1	1	1	0	0	1	0	2	0.14981
**99**	1	0	0	1	1	1	0	0	1	2	0.14981
**100**	1	0	1	0	0	1	0	0	1	2	0.14981
**101**	1	0	1	0	0	1	0	1	0	2	0.14981
**102**	1	0	1	0	1	0	0	1	1	2	0.14981
**103**	1	0	1	0	1	1	0	0	1	2	0.14981
**104**	1	0	1	1	0	0	1	1	1	2	0.14981
**105**	1	0	1	1	0	1	0	0	1	2	0.14981
**106**	1	0	1	1	1	0	0	0	0	2	0.14981
**107**	1	0	1	1	1	0	0	0	1	2	0.14981
**108**	1	0	1	1	1	0	0	1	0	2	0.14981
**109**	1	0	1	1	1	0	1	0	0	2	0.14981
**110**	1	0	1	1	1	0	1	0	1	2	0.14981
**111**	1	0	1	1	1	1	0	1	1	2	0.14981
**112**	1	0	1	1	1	1	1	0	1	2	0.14981
**113**	1	1	0	0	0	0	1	1	0	2	0.14981
**114**	1	1	0	0	0	0	1	1	1	2	0.14981
**115**	1	1	0	0	0	1	0	0	1	2	0.14981
**116**	1	1	0	0	0	1	0	1	0	2	0.14981
**117**	1	1	0	0	0	1	1	1	1	2	0.14981
**118**	1	1	0	0	1	0	0	0	1	2	0.14981
**119**	1	1	0	0	1	0	1	0	0	2	0.14981
**120**	1	1	0	0	1	1	0	0	1	2	0.14981
**121**	1	1	0	0	1	1	0	1	1	2	0.14981
**122**	1	1	0	1	0	0	1	0	0	2	0.14981
**123**	1	1	1	0	0	0	1	1	1	2	0.14981
**124**	1	1	1	0	0	1	0	1	1	2	0.14981
**125**	1	1	1	0	0	1	1	1	0	2	0.14981
**126**	1	1	1	0	1	0	0	0	0	2	0.14981
**127**	1	1	1	0	1	0	0	1	0	2	0.14981
**128**	1	1	1	0	1	0	1	1	0	2	0.14981
**129**	1	1	1	1	0	0	0	1	1	2	0.14981
**130**	1	1	1	1	1	0	0	0	0	2	0.14981

© 2026 Yu L et al. *JAMA Network Open*.

Obs	ADNC	LB	LATE	HS	Macroscopic infarcts	Microinfarcts	CAA	Atherosclerosis	Arteriolosclerosis	N	%
131	1	1	1	1	1	0	0	0	1	2	0.14981
132	0	0	0	0	0	0	1	1	0	3	0.22472
133	0	0	0	0	0	1	0	1	0	3	0.22472
134	0	0	0	0	0	1	1	0	1	3	0.22472
135	0	0	0	0	1	0	0	0	1	3	0.22472
136	0	0	0	0	1	0	1	0	0	3	0.22472
137	0	0	0	0	1	1	1	0	0	3	0.22472
138	0	0	0	0	1	1	1	0	1	3	0.22472
139	0	0	0	0	1	1	1	1	1	3	0.22472
140	0	0	1	0	0	0	0	1	1	3	0.22472
141	0	0	1	0	1	0	0	1	0	3	0.22472
142	0	0	1	0	1	1	0	1	1	3	0.22472
143	0	0	1	1	0	0	0	0	0	3	0.22472
144	0	0	1	1	0	0	0	0	1	3	0.22472
145	0	1	0	0	0	1	0	0	0	3	0.22472
146	0	1	1	1	0	0	0	0	0	3	0.22472
147	1	0	0	0	0	1	1	1	1	3	0.22472
148	1	0	1	0	0	1	0	1	1	3	0.22472
149	1	0	1	0	0	1	1	0	1	3	0.22472
150	1	0	1	0	1	0	1	1	1	3	0.22472
151	1	0	1	1	0	0	0	1	0	3	0.22472
152	1	0	1	1	0	0	1	1	0	3	0.22472
153	1	0	1	1	0	1	1	0	0	3	0.22472
154	1	0	1	1	1	0	1	1	1	3	0.22472
155	1	0	1	1	1	1	1	1	0	3	0.22472
156	1	1	0	0	0	0	0	1	0	3	0.22472
157	1	1	0	0	0	0	0	1	1	3	0.22472
158	1	1	0	0	1	0	0	1	1	3	0.22472
159	1	1	0	0	1	0	1	1	0	3	0.22472
160	1	1	0	0	1	1	0	1	0	3	0.22472
161	1	1	0	0	1	1	1	0	1	3	0.22472
162	1	1	0	0	1	1	1	1	0	3	0.22472
163	1	1	1	0	1	0	0	1	1	3	0.22472

© 2026 Yu L et al. *JAMA Network Open*.

Obs	ADNC	LB	LATE	HS	Macroscopic infarcts	Microinfarcts	CAA	Atherosclerosis	Arteriolosclerosis	N	%
164	1	1	1	0	1	1	0	1	0	3	0.22472
165	1	1	1	0	1	1	1	1	1	3	0.22472
166	1	1	1	1	0	0	1	1	0	3	0.22472
167	1	1	1	1	0	1	0	0	1	3	0.22472
168	0	0	0	0	0	0	1	0	1	4	0.29963
169	0	0	0	0	0	1	0	1	1	4	0.29963
170	0	0	0	0	1	1	0	1	0	4	0.29963
171	0	0	0	0	1	1	1	1	0	4	0.29963
172	0	0	1	0	0	0	0	1	0	4	0.29963
173	0	0	1	0	0	0	1	0	0	4	0.29963
174	0	0	1	0	0	1	0	0	0	4	0.29963
175	0	0	1	0	1	1	0	0	1	4	0.29963
176	0	0	1	0	1	1	0	1	0	4	0.29963
177	0	1	0	0	0	0	1	0	1	4	0.29963
178	0	1	0	0	1	0	0	1	1	4	0.29963
179	0	1	0	0	1	1	0	1	1	4	0.29963
180	1	0	0	0	0	1	0	1	0	4	0.29963
181	1	0	0	0	0	1	0	1	1	4	0.29963
182	1	0	0	0	1	1	1	1	0	4	0.29963
183	1	0	1	0	0	1	1	1	0	4	0.29963
184	1	0	1	0	1	1	0	0	0	4	0.29963
185	1	1	0	0	0	1	1	0	0	4	0.29963
186	1	1	0	0	0	1	1	0	1	4	0.29963
187	1	1	1	0	0	0	0	0	1	4	0.29963
188	1	1	1	0	0	0	0	1	0	4	0.29963
189	1	1	1	0	0	0	0	1	1	4	0.29963
190	1	1	1	0	1	1	0	1	1	4	0.29963
191	1	1	1	1	1	0	1	0	0	4	0.29963
192	0	0	0	0	0	1	1	0	0	5	0.37453
193	0	0	0	0	1	0	0	1	0	5	0.37453
194	0	0	1	0	1	0	0	0	0	5	0.37453
195	0	0	1	0	1	0	0	1	1	5	0.37453
196	0	0	1	0	1	1	0	0	0	5	0.37453

© 2026 Yu L et al. *JAMA Network Open*.

Obs	ADNC	LB	LATE	HS	Macroscopic infarcts	Microinfarcts	CAA	Atherosclerosis	Arteriolosclerosis	N	%
197	0	1	0	0	1	1	0	0	0	5	0.37453
198	1	0	0	0	0	1	1	0	1	5	0.37453
199	1	0	0	0	1	0	0	1	0	5	0.37453
200	1	0	0	0	1	1	1	0	1	5	0.37453
201	1	0	1	0	1	0	0	1	0	5	0.37453
202	1	0	1	0	1	0	1	1	0	5	0.37453
203	1	0	1	1	0	0	0	0	0	5	0.37453
204	1	1	0	0	1	0	0	1	0	5	0.37453
205	1	1	0	0	1	0	1	1	1	5	0.37453
206	1	1	0	0	1	1	1	1	1	5	0.37453
207	1	1	1	1	0	0	0	0	0	5	0.37453
208	1	1	1	1	0	0	0	0	1	5	0.37453
209	1	1	1	1	0	0	1	0	0	5	0.37453
210	0	1	0	0	1	0	0	0	0	6	0.44944
211	1	0	0	0	0	1	1	1	0	6	0.44944
212	1	0	0	0	1	1	1	1	1	6	0.44944
213	1	0	1	0	0	0	0	0	1	6	0.44944
214	1	0	1	0	0	0	1	1	0	6	0.44944
215	1	0	1	0	0	1	1	1	1	6	0.44944
216	1	0	1	0	1	0	0	0	1	6	0.44944
217	1	0	1	0	1	1	1	1	0	6	0.44944
218	1	0	1	1	0	0	1	0	1	6	0.44944
219	1	1	0	0	0	0	0	0	1	6	0.44944
220	0	0	0	0	0	1	0	0	1	7	0.52434
221	0	0	0	0	1	1	0	0	1	7	0.52434
222	0	0	1	0	0	0	0	0	1	7	0.52434
223	0	1	0	0	0	0	0	0	1	7	0.52434
224	1	0	0	0	1	0	0	0	1	7	0.52434
225	1	0	0	0	1	1	0	1	0	7	0.52434
226	1	0	1	1	0	0	1	0	0	7	0.52434
227	1	1	0	0	1	1	0	0	0	7	0.52434
228	1	1	0	0	1	1	1	0	0	7	0.52434
229	1	1	1	0	0	1	0	0	0	7	0.52434

© 2026 Yu L et al. *JAMA Network Open*.

Obs	ADNC	LB	LATE	HS	Macroscopic infarcts	Microinfarcts	CAA	Atherosclerosis	Arteriolosclerosis	N	%
230	1	1	1	0	0	1	1	0	0	7	0.52434
231	0	1	1	0	0	0	0	0	0	8	0.59925
232	1	0	0	0	0	1	0	0	1	8	0.59925
233	1	0	1	0	0	0	0	1	0	8	0.59925
234	1	0	1	0	0	0	0	1	1	8	0.59925
235	1	0	1	0	0	0	1	1	1	8	0.59925
236	1	0	1	0	1	0	0	0	0	8	0.59925
237	1	0	1	0	1	1	0	1	0	8	0.59925
238	1	0	1	0	1	1	1	0	0	8	0.59925
239	1	1	1	0	0	0	1	0	1	8	0.59925
240	1	1	1	0	1	1	1	0	0	8	0.59925
241	1	0	0	0	0	0	1	1	1	9	0.67416
242	1	0	0	0	1	0	1	0	1	9	0.67416
243	1	0	0	0	1	1	0	0	1	9	0.67416
244	1	0	0	0	1	1	1	0	0	9	0.67416
245	1	0	1	0	0	1	1	0	0	9	0.67416
246	1	0	1	0	1	1	1	0	1	9	0.67416
247	1	0	1	0	1	1	1	1	1	9	0.67416
248	1	1	0	0	1	0	0	0	0	9	0.67416
249	0	1	0	0	0	0	0	1	0	10	0.74906
250	1	0	0	0	1	0	1	1	0	10	0.74906
251	1	0	1	0	1	1	0	1	1	10	0.74906
252	1	0	0	0	0	0	1	1	0	11	0.82397
253	1	0	0	0	1	0	1	1	1	11	0.82397
254	1	0	1	0	1	0	1	0	0	11	0.82397
255	1	1	0	0	0	0	1	0	1	11	0.82397
256	0	0	0	0	1	1	0	1	1	12	0.89888
257	1	0	0	0	0	0	0	1	1	12	0.89888
258	1	0	0	0	1	0	0	1	1	12	0.89888
259	1	0	0	0	0	1	1	0	0	13	0.97378
260	1	0	1	0	0	0	1	0	1	13	0.97378
261	1	0	1	0	0	1	0	0	0	13	0.97378
262	1	1	0	0	0	1	0	0	0	13	0.97378

© 2026 Yu L et al. *JAMA Network Open*.

Obs	ADNC	LB	LATE	HS	Macroscopic infarcts	Microinfarcts	CAA	Atherosclerosis	Arteriolosclerosis	N	%
263	1	0	0	0	1	1	0	1	1	14	1.04869
264	0	0	0	0	0	0	0	1	1	15	1.12360
265	0	0	0	0	1	0	0	1	1	15	1.12360
266	0	0	0	0	1	1	0	0	0	15	1.12360
267	1	0	0	0	1	1	0	0	0	15	1.12360
268	1	0	0	0	0	0	1	0	1	16	1.19850
269	1	1	1	0	0	0	1	0	0	16	1.19850
270	1	0	0	0	0	0	0	0	1	17	1.27341
271	1	0	0	0	1	0	1	0	0	21	1.57303
272	1	1	1	0	0	0	0	0	0	21	1.57303
273	1	0	0	0	0	0	0	1	0	22	1.64794
274	1	0	0	0	1	0	0	0	0	22	1.64794
275	1	0	0	0	0	1	0	0	0	23	1.72285
276	1	0	1	0	0	0	0	0	0	25	1.87266
277	1	1	0	0	0	0	1	0	0	25	1.87266
278	1	1	0	0	0	0	0	0	0	33	2.47191
279	1	0	1	0	0	0	1	0	0	35	2.62172
280	1	0	0	0	0	0	1	0	0	57	4.26966

© 2026 Yu L et al. *JAMA Network Open*.

eTable 2. Common AD/ADRD Genetic Variants (Dosage) by Latent Neuropathologic Profiles

Genetic variants, Mean (SD)	Profile 1	Profile 2	Profile 3	Profile 4	Profile 5
CR1 **(rs4844610)**	1.63 (0.51)	1.60 (0.59)	1.61 (0.57)	1.69 (0.53)	1.61 (0.55)
BIN1 **(rs6733839)**	0.77 (0.65)	0.79 (0.66)	0.78 (0.60)	0.87 (0.63)	0.78 (0.65)
CD2AP **(rs9473117)**	0.49 (0.58)	0.50 (0.59)	0.57 (0.63)	0.59 (0.65)	0.58 (0.65)
CLU **(rs9331896)**	1.13 (0.67)	1.24 (0.68)	1.14 (0.69)	1.14 (0.71)	1.18 (0.69)
PICALM **(rs3851179)**	1.33 (0.68)	1.22 (0.66)	1.23 (0.72)	1.21 (0.69)	1.30 (0.66)
SORL1 **(rs11218343)**	0.05 (0.22)	0.09 (0.28)	0.08 (0.28)	0.06 (0.24)	0.08 (0.28)
ABCA7 **(rs3752246)**	1.60 (0.51)	1.69 (0.46)	1.64 (0.52)	1.62 (0.48)	1.67 (0.49)
TMEM106B **(rs1990622)**	0.88 (0.73)	0.82 (0.73)	0.79 (0.73)	0.82 (0.72)	0.85 (0.69)

© 2026 Yu L et al. *JAMA Network Open*.

eFigure 1. Burden of Copathologies of the Study Participants

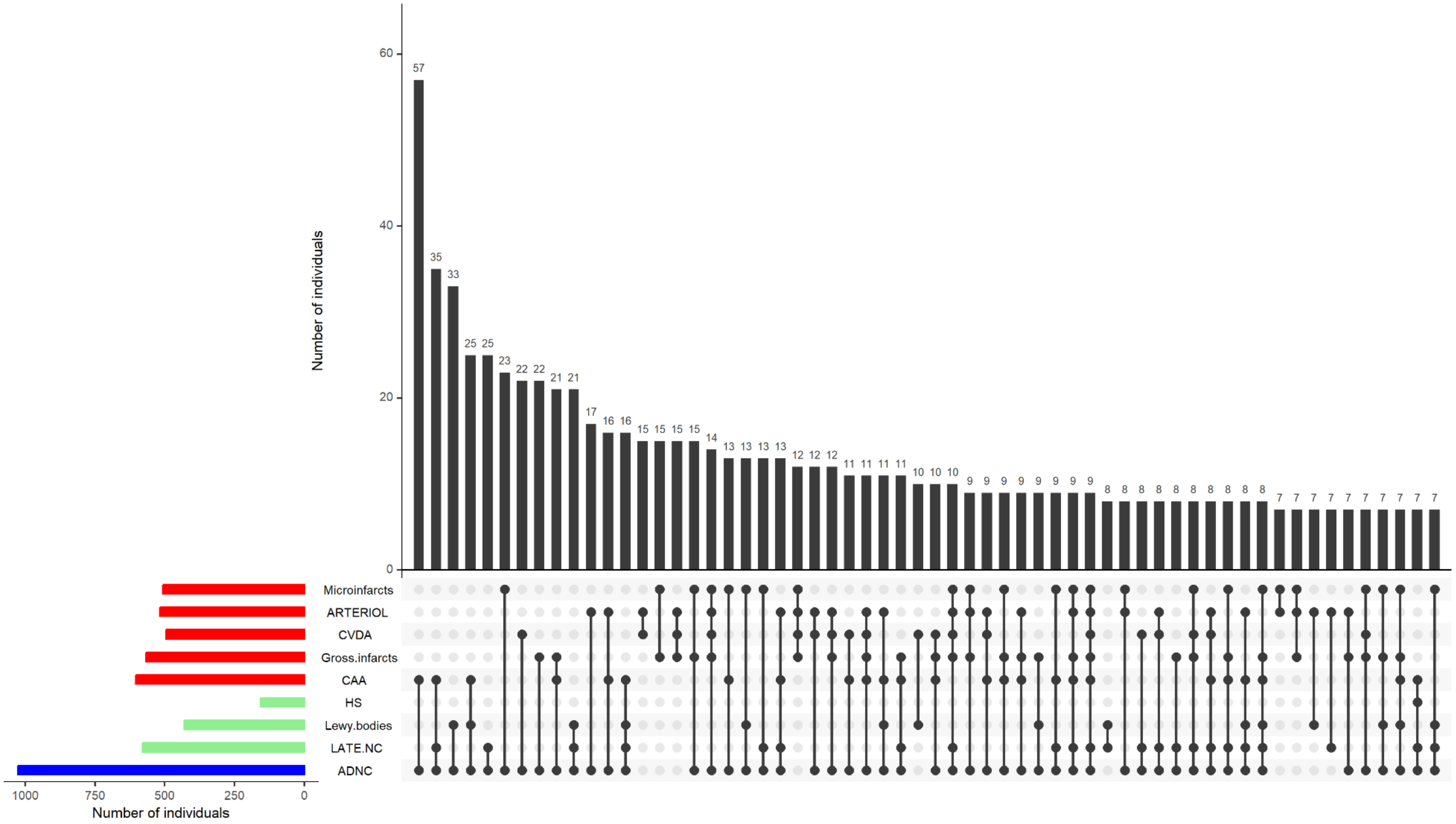

This figure illustrates the burden of mixed pathologies of the study participants. The bar chart on the lower left corner shows the frequencies of individual neuropathologic indices. The connected black dots on the x-axis indicate the specific combination of neuropathologies represented (top 60 combinations shown). The histograms in the main panel show the frequencies of the mixed pathologies, ordered by overall frequency.

© 2026 Yu L et al. *JAMA Network Open*.

eFigure 2. Hierarchical Cluster Dendrogram

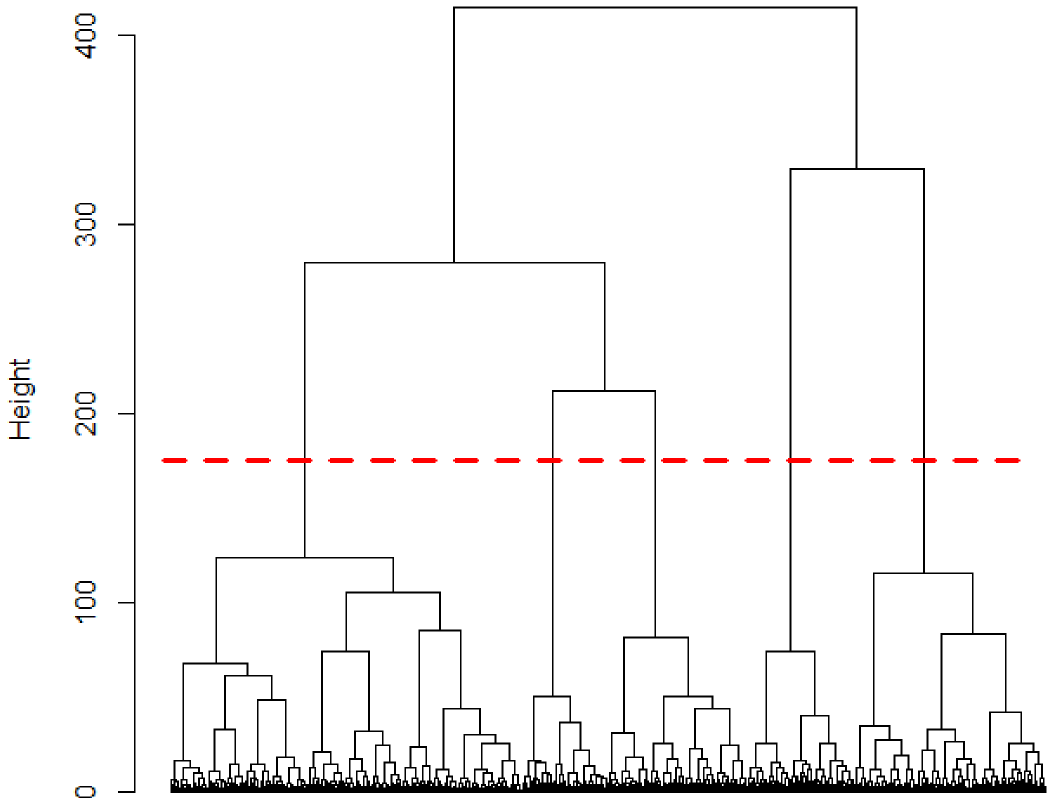

The figure illustrates the clustering that identifies the latent neuropathologic profiles. The height on the y-axis measures the distance between clusters. At a given height, each vertical line represents a cluster. Observations are clustered by drawing a horizontal line through the dendrogram. The optimal number of clusters can be determined by branches with largest reductions in height. The resulting clusters correspond to the five neuropathologic profiles in the results.

© 2026 Yu L et al. *JAMA Network Open*.

eFigure 3. Gap Statistic for Optimal Number of Clusters

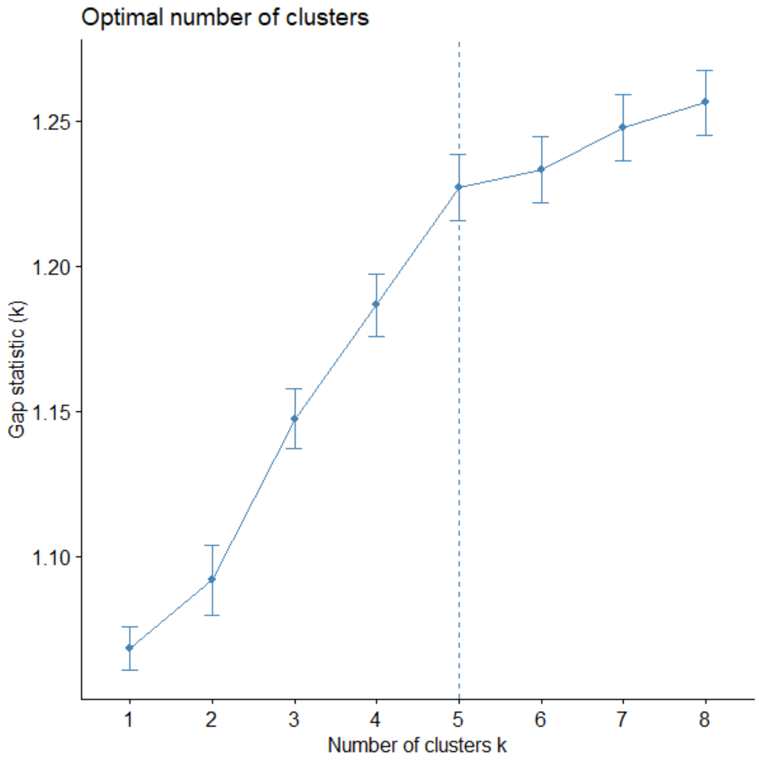

The figure presents Gap statistic for optimal number of clusters. It is evident that the elbow of the change in Gap statistic occurs at 5. The choice of 5 clusters is also supported following a previously proposed criterion (Tibshirani, R., Walther, G. and Hastie, T. (2001). Estimating the number of data clusters via the Gap statistic. Journal of the Royal Statistical Society B, 63, 411–423).

© 2026 Yu L et al. *JAMA Network Open*.
