# Supplementary material for: Neuropathologic Profiles and Associated Cognitive Trajectories in Community-Living Older Adults
Source: JAMA Netw Open. 2026 Jan 16;9(1):e2554354. doi: 10.1001/jamanetworkopen.2025.54354 (PMC12811812; doi:10.1001/jamanetworkopen.2025.54354)
Supplement: Supplement 2. — Data Sharing Statement [file jamanetwopen-e2554354-s002.pdf]

Data Sharing Statement

Yu. Neuropathologic Profiles and Associated Cognitive Trajectories in Community-Living Older Adults. *JAMA Netw Open*. Published January 16, 2026.
doi:10.1001/jamanetworkopen.2025.54354

Data
Data available: Yes
Data types: Participant data with identifiers
How to access data: Data used in the analyses are available by request through the RADC Research Resource Sharing Hub at https://www.radc.rush.edu/.
When available: With publication

Supporting Documents
Document types: None

Additional Information
Who can access the data: Researchers whose proposed use of the data has been approved
Types of analyses: For research purposes only
Mechanisms of data availability: Submission and approval of data request, and a signed data use agreement on file
